# Developing “De-stress pain,” an intervention to reduce chronic pain-related distress: Proof of concept and qualitative assessment of acceptability

**DOI:** 10.1177/20494637261464804

**Published:** 2026-07-03

**Authors:** Stephanie Hughes, Tamar Pincus, Adam W. A. Geraghty, Carolyn A. Chew-Graham, Beth Stuart, Paul Little, Michael Moore, Hollie Birkinshaw

**Affiliations:** 1 Department of Psychology, 7423 University of Southampton, Southampton, UK; 2 Faculty of Medicine, Clinical Neurosciences-Psychiatry, Academic Centre, 7423 University of Southampton, Southampton, UK; 3 School of Medicine, 4212 Keele University, Keele, UK; 4 Wolfson Institute of Population Health, 4617 Queen Mary University of London, London, UK

**Keywords:** chronic pain, distress, depression, intervention development, person-based approach, social prescribers, persistent pain

## Abstract

**Introduction:**

People with persistent musculoskeletal (MSK) pain often experience distress, distinct from depression. Current referral pathways and interventions are suboptimal for this group. We developed and tested the acceptability and proof of concept of *De-Stress Pain*, an intervention to reduce pain-related distress.

**Methods:**

Guided by principles from Acceptance and Commitment Therapy, behavioural activation, and the Person-Based Approach (PBA) De-*Stress Pain* provided 4–6 social prescriber sessions over 12 weeks, plus access to a mental wellbeing website promoting engagement in meaningful and pleasurable activities. Acceptability of the intervention and study procedures was assessed qualitatively using semi-structured interviews with participants and social prescribers before and after the intervention programme.

**Results:**

Sixteen participants were recruited and 11 completed the intervention alongside four social prescribers. Participants described the intervention as acceptable and valued the combination of social prescriber support, accountability, and encouragement to re-engage in meaningful and pleasurable activities. Social prescribers reported that the intervention was acceptable to deliver and aligned with their existing practice, although limited appointment time and participants’ financial constraints could affect engagement. Some participants initially viewed pleasurable activities as indulgent, which acted as a barrier to engagement. Findings also suggested that future iterations may benefit from refining eligibility criteria to better identify individuals experiencing sufficient pain-related distress. Improvements in mood measures and participant reports of increased hope, activity, and wellbeing suggested the intervention showed promise for supporting people with pain-related distress.

**Conclusions:**

This study demonstrates that De-Stress Pain was acceptable to both participants and social prescribers and feasible to deliver within social prescribing services. The findings identified several factors requiring consideration in future iterations, including participant selection, time constraints, and financial barriers to engagement. De-Stress Pain is, to our knowledge, among the few pain-related interventions specifically designed for delivery by social prescribers within primary care settings.

## Introduction

Musculoskeletal (MSK) conditions affect over 20 million people in the UK and are a major contributor to years lived with disability (YLD).^1^ Persistent pain is a common symptom, with back pain being the leading cause of disability. MSK conditions are predominantly managed in primary care, accounting for around one in seven GP consultations, and contribute substantially to work absence and economic inactivity.^2,3^

Depression is four times more common in people with long term pain.^3^ Those with MSK pain report struggling to live well.^4^ Plans are difficult to make due to uncertainty, physical demands, and fatigue; relationships become strained, and work is challenging. Many feel like a burden, live with guilt and low mood, and withdraw from activities.^5,6^ Individuals also report frustration and disillusion with medical support, both for their pain and their low mood.^6^ Doctors in turn have told us that it can be difficult to help these patients.^6^ Guidelines recommend antidepressants for low mood and/or persistent pain,^7,8^ but these are not always helpful or acceptable to patients.^9^ Previous research has highlighted patient reservations about referrals to health care providers with expertise in mental health, for example, NHS Talking Therapy Services and brief interventions with Psychological Well-being Practitioners offering predominantly cognitive behavioural therapy and behavioural activation. Patients felt this invalidated their pain, indicating ‘it was all in their head’.^6^ Even after completing pain management courses, which typically emphasise self-management, some patients report finding themselves struggling as the support ends.^10^

Many people living with persistent pain experience pain-related distress. This is a specific form of psychological distress arising from difficulty coping with pain functioning as interruptive stressor, impacting on everyday life. While it can overlap with symptoms of depression, it is conceptually distinct.^11^ Having to withdraw from cherished activities because of pain can cause distress and threaten self-identity, so that over time a person’s sense of self becomes closely tied to their experience of pain.^12^ Our research focuses on developing an intervention to reverse this enmeshment.

A combination of validated, evidence-based psychological theories refined our approach. One approach to improving wellbeing and reducing the extent to which pain dominates a person’s sense of self is Acceptance and Commitment Therapy (ACT), based on the premise of living life to the full amidst the pain.^13^ A recent overview of systematic reviews of ACT interventions suggests that it is effective at reducing depression and increasing function and wellbeing.^14^

ACT is an action-oriented approach requiring patients to commit to making changes to bring them closer to their desired outcomes.^15^ ACT is particularly appropriate in the context of pain-related distress because it focuses on helping individuals engage in meaningful activities and improve psychological flexibility despite ongoing pain.

We were also guided by self-determination theory,^16^ which suggests interventions targeting behaviour change can be improved by intervention processes supporting needs for autonomy, competence, and relatedness. That is, within the context of an intervention one needs to feel: in control of behaviours and goals (autonomy); confident one has the skills needed for success (competence); and a sense of belonging and attachment to others (relatedness or connection). A recent systematic review with meta-analysis suggests that interventions based on self-determination theory are more likely to result in improvements to psychological and physical wellbeing than usual care, and of importance, result in changes in behaviour.^17^

Finally, we were interested in incorporating principles from behavioural activation in our approach. Behavioural activation is considered a parsimonious intervention, based on simple and highly acceptable behavioural therapy principles that use activity scheduling to reconnect individuals with society and their environment.^18^ Such interventions have been found to be effective for people living with depression. In the context of pain-related distress, value-aligned pleasant activity scheduling, central in behavioural activation,^19^ reflects an additional evidence-based approach, congruent with ACT and self-determination theory (SDT).

Overall, our aim was to develop a patient-centred intervention to support those experiencing pain-related distress. Intervention development was guided by the person-based approach (PBA),^20^ an established framework that uses qualitative research to ensure interventions are grounded in the perspectives, psychosocial context, needs and preferences of the target user group, including the challenges, barriers, and facilitators influencing behaviour change and intervention engagement.^21^

The way in which social prescribers work aligns with our theoretical underpinnings in trying to reduce enmeshment with pain, that is, specifically trying to orient people away from their pain experience, to build other aspects of their lives. To have worked with pain specialists (physiotherapists, psychologists) would have been counter to the core of this intervention.

We conducted a proof-of-concept (POC) study to test the intervention’s acceptability. The aim of a POC study is to explore whether the intervention could work as intended, rather than then to prepare for full randomised controlled trials. POC studies aim to determine whether an approach is feasible and worthy of further development, while identifying potential issues and refining study design before pilot and real-world testing.^22^

## Methods

### Patient and public engagement and involvement

Our Patient and Public Involvement and Engagement (PPIE) group comprised four individuals with lived experience of persistent pain. An initial meeting was held to introduce the aims of the project, discuss expectations, and agree ways of working. PPIE contributors were involved throughout intervention development and refinement, including reviewing intervention materials, study procedures, and participant-facing documents. Meetings were planned approximately every 2–3 months; however, a flexible and responsive approach was adopted, with additional discussions held as required during key stages of project development. Meetings typically lasted approximately 45–60 min. Input from the group was considered sufficient when no substantially new issues or recommendations relating to intervention content, study procedures, or participant materials were being identified during discussions.

## Methods for intervention development

**Figure 1.**
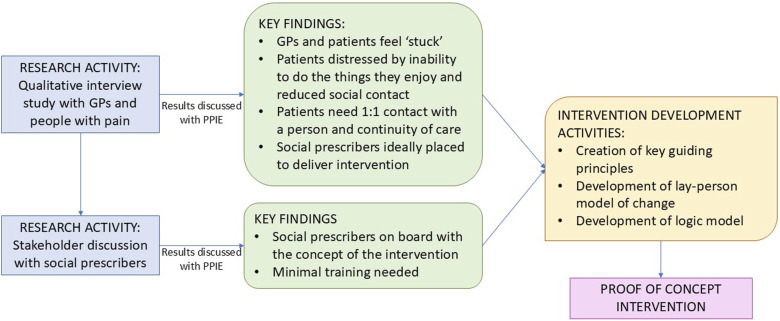
Flow chart to show intervention planning and development process.

We commenced our target user studies with interviews with GPs and people with pain, and used the results to guide our next activity, which was a stakeholder discussion with social prescribers. We discussed our findings with the study PPIE group and worked with them to develop a lay-person model of change. We used the findings from these activities to create key guiding principles and a logic model (see Figure 1 below).

### Background

In work leading to this study, published elsewhere,^6^ semi-structured interviews were conducted with people with persistent pain (*n* = 21) and General Practitioners (*n* = 21) to explore perspectives and understanding of primary care management of people with persistent MSK and distress.

The findings from these interviews were discussed with our PPIE group, and provide the foundations for the De-Stress *Pain* intervention (Table 1). See appendix 1 for quotes to support the findings outlined in Table 1.

**Table 1.** Foundations of the intervention resulting from interview findings and informed by PPIE.

Findings	Intervention implications
General practitioners felt “stuck” when dealing with pain-related distress; patients reported dissatisfaction from general practice options.	• Delivery outside of medical setting. • Not delivered by GPs.
GPs recognise the difference between depression and pain-related distress,	• Recruitment through primary care is viable.
GPs considered that social prescribers are well-placed and have time to encourage social reactivation, .	• Delivery by social prescribers would fit within NHS systems and address intervention principles.
Patients agreed strongly that a person delivering one-to-one contact was essential. The mode of contact mattered less.	• Participants need 1:1 contact with a person.
There was support for accessible online information in addition to person-based delivery	• Create a simple, user-friendly website, ensure use of lay language, images, and ease of navigation.
Patients and PPIE told us that the most distressing aspects of living with pain were inability to do the things you used to enjoy, reduced social contact, and difficulty making plans.	• Base the intervention on enjoyable activities. • Allow for opportunities to carry out some activities with other people. • Allow flexibility around approaching activities (no schedule, no use of the word “should”).
Addressing pain-related distress required several consultations and continuity of care is important	• Offer more than a single session, and ensure sessions are with the same person.
Moving the focus away from pain is helpful	• Don’t focus on pain.
Credibility is important	• Intervention needs to be delivered by experts in social reactivation.

### Stakeholder discussion with social prescribers

Our exploration of the needs of people with pain suggested social prescribers may be well-placed to deliver an intervention for this population. Stakeholder discussions with social prescribers (SPs) were conducted as part of the intervention development process and are therefore reported here. To further explore this idea, we held a 60-min group online stakeholder discussion with 9 SPs. The SPs were part of a charitable organisation working across The Midlands in the UK. SPs had no prior knowledge of the proposed intervention and were not required to complete any preparatory tasks. To facilitate our understanding, we asked questions about what they do in their role, how many sessions they typically offer, whether they conduct these face-to-face or by telephone, whether they currently see people with pain-related distress, and the challenges they face. We then explored their thoughts about our intervention specifically for pain-related distress being delivered by a social prescriber and whether they felt this was something they could deliver, and the challenges that they might face. Our main findings are presented below in Table 2. At the request of the partner charity, this discussion was not audio-recorded. Two members of the research team took detailed contemporaneous notes during the session, which were compared and expanded immediately afterwards to enhance accuracy and minimise recall bias. Direct participant quotations were therefore unavailable.

**Table 2.** Stakeholder discussion suggestions and implications for the intervention.

Stakeholder insight	Influence on intervention design
Social prescribing is tailored to individual needs.	The intervention needs to adjust to participant needs and circumstances.
Social prescribers try to give clients ownership over decisions and encourage independence.	The intervention should support participants to sustain changes beyond the intervention period.
Social prescribers can give more time to the clients compared to other health care professionals.	This supports the rationale for selecting social prescribing to deliver the intervention
The number of social prescriber sessions is usually tailored to client need.	The intervention should incorporate flexibility within a defined intervention period and session range.
Social prescriber sessions are offered either face-to-face or by telephone, and this flexibility is valuable to their clients.	The intervention should offer the flexibility of telephone or face-to-face.
Social prescribers described referrals with limited scope around pain management	The intervention should focus on mood, wellbeing, and meaningful activity alongside persistent pain.
Social prescribers identified client transport (cost and availability) as a barrier.	The intervention needs to offer “at home” options and consider transport-independent ways of getting out of the home
Social prescribers feel familiar, comfortable, and qualified to deliver the intervention	Minimal training required.
Social prescribers have knowledge of the local area including transport systems, local community groups/activities, and personal contacts with key people in community groups.	Local knowledge is necessary

### Intervention development

Informed by the PBA framework, findings from the exploratory work were used to develop a lay-person model of change, Key Guiding Principles, and a logic model.

## Lay-person’s change model developed with PPIE

**Figure 2.**
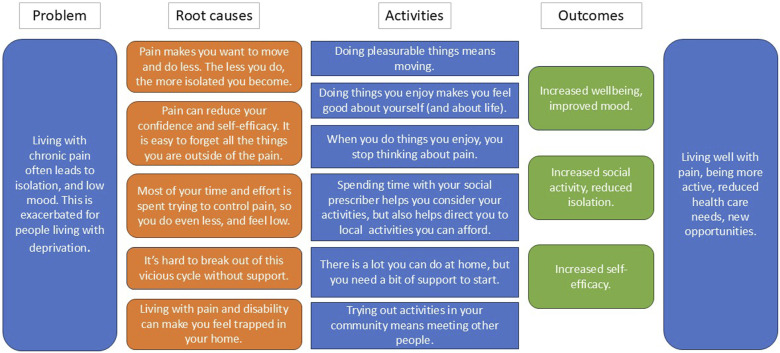
Lay-person’s model of change.

After our discussion with social prescribers, we came back to our PPIE group to collaboratively develop a model to demonstrate their opinion on how change would occur within De-Stress Pain (Figure 2).

## Key Guiding Principles

Key Guiding Principles summarise the design objectives and how they will be achieved. These principles focus decision-making throughout the development phase,^21^ see Table 3 below.

**Table 3.** Key guiding principles.

User context	Key design objective	Intervention
People with pain suffer low mood.	Ensure users understand the difference between clinical depression and pain-related distress.	Provide clear explanation about the difference between clinical depression and pain-related distress.
People with pain have lost their identity.	Help users re-find their sense of self.	Use the video ‘pain and me’^23^ to communicate the message ‘you are bigger than the pain’. Encourage users to compartmentalise pain so it is a PART of them, rather than ALL of them. Shift focus onto the parts not impacted by pain.
People with pain have stopped engaging with the things they love.	Encourage social reactivation.	Offer suggestions of enjoyable activities
People with pain focus on pain.	Clear rationale about why shifting focus onto living well will be beneficial.	Focus on how an individual’s life/psychological wellbeing can be improved.

## Refining the intervention with psychological theory

The main premises of Acceptance Commitment Therapy (ACT) and Self-Determination Theory were considered when refining intervention design. Table 4 below describes the implications of these theories for our intervention.

**Table 4.** Psychological theory and our intervention.

Theory	Premise	Implications for intervention
Acceptance commitment therapy (ACT)	ACT helps people with pain accept their emotions and focus on their personal values.	A shift from a focus on pain to positive, pleasurable activities.
Self-determination theory	Wellbeing can be improved by meeting needs for autonomy, competence and relatedness: Feeling in control, confident in one’s abilities, and connected to others.	Participants should be supported to create a personalised, achievable plan based on their preferences, helping them feel in control. Plans should consider practicalities (e.g. equipment or travel needs) and encourage social activities to enhance connection with others.

## Logic model

**Figure 3.**
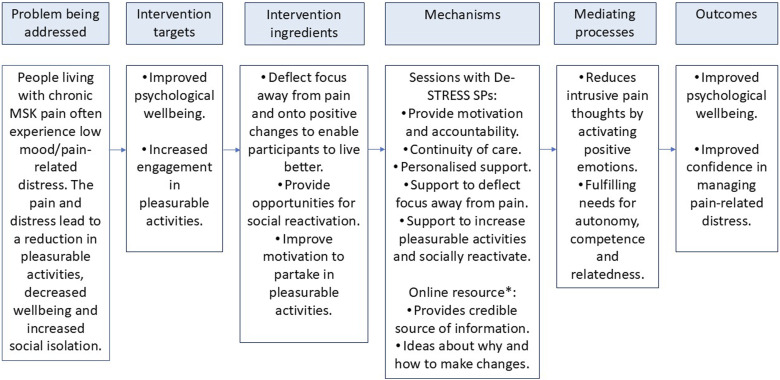
Logic model. *”Online resource” refers to a mental wellbeing website for participants, promoting engagement in meaningful and pleasurable activities.

As recommended by the MRC complex intervention guidelines,^24^ we developed a logic model to outline the hypothesised causal mechanisms involved in bringing about change. Figure 3 below shows the logic model for how our intervention would bring about change in people with pain-related distress according to our development work.

## The intervention

The intervention planning and development phases resulted in a proof of concept intervention. The De-Stress Pain intervention was designed to offer 4–6 sessions with a social prescriber (named “De-Social Prescribers (SPs)” for the purposes of the intervention) over 12 weeks. Drawing upon information obtained in our background discussions with social prescribers we decided 4–6 sessions was the most appropriate amount of contact to allow for participants to create and adjust personal plans where needed. The De-Stress SPs received 3 h online training with the research team and a manual (developed by the research team) to guide them through sessions. The training and accompanying manual covered the background and rationale for the intervention, a detailed description of the social prescriber role and required activities, guidance on working with individuals experiencing pain-related distress, and safety procedures to follow if concerns about a participant arose. Participants were offered access to a study website (developed by the research team) which included modules about self-compassion, the rationale behind increasing pleasant activities, and provided suggestions for local activities that participants might like to try. The list of suggested local activities was developed with PPIE and included walking groups, painting, pottery, swimming, online film clubs, and book clubs. The De-Stress SP sessions aimed to:

• Help participants identify accessible pleasurable activities and create a plan to carry them through.
• Identify ways to adapt activities when needed (e.g. when experiencing pain flare-up).
• Deflect conversations about pain and encourage participants to focus their energy on positive changes they can make.
• Support participants if their plans need changing.

**Figure 4.**
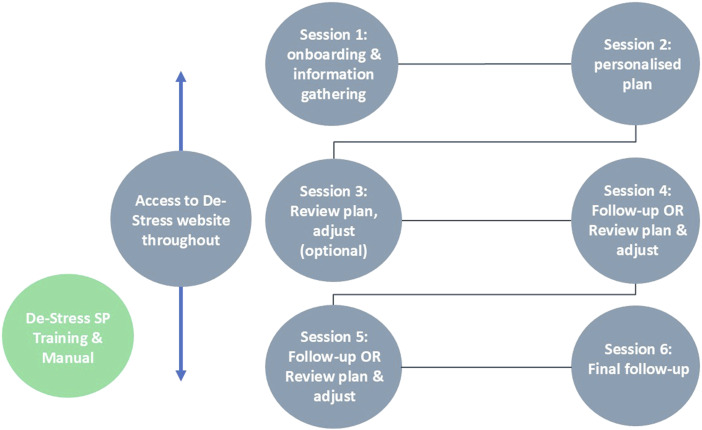
The intervention.

Figure 4 below outlines the intervention sessions.

The intervention was tested for acceptability in a proof-of-concept study.

## Methods for proof-of-concept and acceptability study

### Aim

To test the acceptability and proof of concept of an intervention to reduce pain-related distress in people with persistent MSK pain.

### Design

A repeated measures mixed-methods proof of concept study consisting of a quantitative survey and qualitative interviews. Both quantitative measures and qualitative interviews were completed at baseline and 12 weeks post baseline.

### Participants

#### Eligibility criteria

Participants were eligible if they were aged 18 years or over, experienced persistent musculoskeletal (MSK) pain lasting longer than 3 months (e.g. knee, hip, or back pain, or fibromyalgia), were able to complete questionnaires and interviews in English, and were able to provide informed consent. Participants were excluded if they lacked capacity to provide informed consent, indicated suicidal ideation, had pain associated with malignant disease as reported in GP notes, or did not have access to the internet.

#### Setting and recruitment

This aspect of the study was set in a primary care context, with patients and Social Prescribers recruited via primary care practices and primary care Clinical Research Networks respectively.

Specifically, Social Prescribers were recruited as De-Stress SPs via Clinical Research Network mailouts, and the BNSSG (Bristol, North Somerset, and South Gloucestershire) Training HuB.

People with persistent pain were identified through 3 GP practices (one in Cambridgeshire, one in Berkshire, and one in Stoke-on-Trent). These practices responded to expression of interest to be included in the study, and also had social prescribers at the practice that could take part. Practices searched patient databases using relevant Systematised Nomenclature of Medicine (SNOMED) codes. Potential participants were posted an invite letter and Participant Information Sheet containing an online link to the screening questionnaire and consent form. Participants consented online.

Confidentiality was maintained throughout recruitment, with GP practices distributing invitation materials directly to potentially eligible participants. The research team only accessed identifiable information from individuals who chose to contact the study team and provide informed consent. Study data were stored securely on password-protected university systems in accordance with data protection regulations.

Potential participants completed a screening questionnaire before proceeding to the baseline questionnaire. The screening questionnaire ensured they met the eligibility criteria described above, and that they were not experiencing suicidal thoughts. If a potential participant was not eligible they were informed immediately. Details of those who indicated they were experiencing suicidal thoughts were passed onto their GP for follow-up.

## Quantitative outcome measures

All questionnaires were completed online. Those who passed screening were given instant access to the baseline questionnaire. A follow-up questionnaire was issued at 12 weeks post baseline. Table 5 below describes the measures and the timepoint(s) they were completed. There is currently no standardised validated measure specifically designed to assess pain-related distress. Therefore, to capture this construct, participants rated their pain-related distress on a 0–10 scale, as described in Table 5. In addition, proxy measures relevant to psychological distress and wellbeing, including the 4DSQ and single-item questions, were selected to provide an initial indication of potential changes associated with the intervention. These measures were considered appropriate for this proof-of-concept study given its exploratory focus and the absence of an established pain-related distress outcome measure.

**Table 5.** Measures included in the survey.

Variable	Measure	Baseline or 12 week follow-up?
Clinical and demographic characteristics	Type of musculoskeletal pain, age, gender, education, ethnicity, employment status.	Baseline only
Mood	Single item to distinguish distress due to pain, due to other things, or both. (Over the last 4 weeks have you experienced distress (low mood/depression/anxiety)? Answer: Yes due to my pain/Yes due to other reasons/Yes due to my pain and other reasons/No)	Baseline only
	Single item rating distress 0–10 over last 2 weeks (Over the last 2 weeks, on average, how much distress have you been experiencing because of your pain? Answer: 0 (no distress)–10 (extreme distress)	Baseline and follow-up
	DAPOS, 11-item scale to assess depression, anxiety and positive outlook.^25^	Baseline and follow-up
	WEMWBS, 14-item scale to assess wellbeing.^26^	Baseline and follow-up
	4DSQ, four subscales measuring: distress (16 items), depression (6 items), anxiety (12 items), and somatization (16 items).^27^	Baseline and follow-up
Pain chronicity	Single item question to assess chronicity (how long since no pain for a month).^28^	Baseline only
	Single item asking number of troublesome days in pain in the last 4 weeks.^29^	Baseline and follow-up
Pain intensity	Numerical rating scales rating worst, least, average, and current pain, and coping.^30^	Baseline and follow-up
Musculoskeletal health	MSK-HQ, 15-item scale to assess quality of life and pain interference in patients with MSK pain.^31^	Baseline and follow-up

## Interviews with participants

Semi-structured interviews with participants were conducted at two timepoints (baseline and 12 weeks post baseline) using an iterative topic guide.

- Baseline interview: Conducted after the participant completed the baseline questionnaire, and before they started the intervention. This interview gathered insight into the participant’s context, their experiences of pain-related distress, why they wanted to be part of the study and their expectations of participation.
- Follow-up interview: Conducted after the participant completed all sessions with their De-Stress SP and the quantitative questionnaire. The interview explored their experiences of the intervention, any changes they had made since taking part, and any barriers or facilitators to engaging with the intervention they encountered.

## Interviews with De-stress social prescribers

Semi-structured interviews were conducted with the De-Stress SPs at the end of their involvement in the study. These interviews explored overall views of providing support as part of the study, perceptions of participant experiences, the relationships they built with participants, their views about the training and manual provided as part of the study, and their thoughts on limitations and future research.

## Sample size

As a small study to assess the acceptability and proof of concept of the intervention, a formal sample size calculation was not appropriate. In line with other intervention development and proof of concept studies in similar populations^32,33^ we set a recruitment target of 20 to explore a range of experiences, this number has previously been sufficient to identify core issues ahead of further feasibility work and trials.

## Analysis

For quantitative analysis we focused primarily on descriptive data regarding the recruitment process, numbers recruited, withdrawn and lost to follow-up. We also describe the number of sessions delivered by the social prescribers and the period over which they were delivered. We present data on participant characteristics. Descriptive statistics including means and standard deviations for possible clinical outcomes that may be used in future trials are also presented. We do not present tests for statistical significance; we did not power for hypothesis testing.

Interviews were transcribed verbatim, imported into NVivo and analysed by authors HB and SH using reflexive thematic analysis (Braun & Clarke 2006). The analysis was primarily inductive, allowing themes to be generated from the data rather than applying a pre-existing coding framework. Both researchers independently read and re-read transcripts to ensure familiarity, before undertaking initial coding. Coding was then compared and discussed, with discrepancies resolved through discussion to develop a shared coding framework. Codes were subsequently grouped into broader themes, with patterns, relationships, and overarching narratives explored iteratively. Theme development was refined through regular discussions between HB and SH and further reviewed within the wider research team. Input from the PPI group contributed to interpretation and helped ensure relevance and credibility of the findings.

The analysis took a pragmatic approach, focusing on participants’ experiences of the intervention and their practical implications.

**Figure 5.**
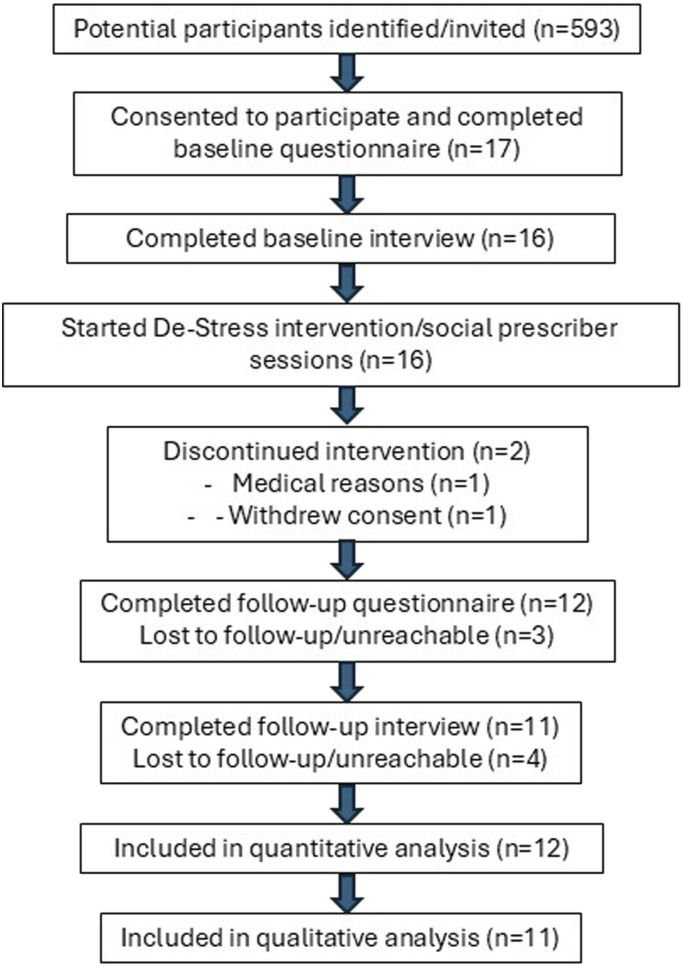
Flow chart of participants.

## Results

Figure 5 shows the flow or participants through the study.

Four De-Stress SPs were recruited working across 3 primary care practices. 593 potential participants were sent an invitation letter with details about the study. 17 participants consented to the study and completed the baseline questionnaire, and 12 participants completed the follow-up questionnaire; 1 withdrew for medical reasons, 1 withdrew consent, and 3 were unreachable. 16 participants participated in the baseline interview, and 11 completed the follow-up interview. All 4 De-Stress SPs took part in a qualitative interview. Participants were recruited between 01/09/2023 and 31/12/2023. Follow-up data collection was complete by 21/04/2024.

### Participant characteristics

Recruited participants were mostly white (*n* = 16), female (*n* = 12), and ages ranged 24–79, with a mean age of 53.2. The employment status of participants was mixed, full-time (*n* = 7), part-time (*n* = 2), retired (*n* = 5), and unemployed (*n* = 3). Participants presented with a range of pain conditions; some had pain in multiple sites due to multiple reasons. Back (*n* = 8) and knee pain (*n* = 4) were most common. Four reported arthritis and two reported fibromyalgia. Pain chronicity varied with some living with pain for more than 10 years (*n* = 6), 5–10 years (*n* = 5), 3–5 years (2), 1–2 years (*n* = 3), and 7–12 months (*n* = 1). With no standardised measure of pain-related distress it is not possible to classify participants by severity, however, participants rated their pain-related distress on a scale of 0–10, participants had a mean score of 6.6, range of 4–10, median of 7, and mode of 7.

### Survey results

Table 6 below shows the mean scores and standard deviations for each of the measures at baseline and 12-week follow-up. Although the study was not powered to assess significance and meaningful conclusions cannot be drawn, administering this survey was nonetheless valuable in highlighting potential issues with data collection and ensuring the feasibility of the measures for future research. There were no issues with data collection, and measures were fully completed, thus deemed feasible for use in future studies.

**Table 6.** Mean scores and standard deviations at baseline and 12-week follow-up.

Scale	Direction of improvement	Baseline (*n* = 17) Mean (SD)	Follow-up (*n* = 12) Mean (SD)
Pain intensity	Lower	5.18 (1.33)	5.58 (1.62)
Pain coping	Higher	4.94 (1.71)	6.08 (1.88)
Distress due to pain	Lower	6.60 (1.62)	5.25 (2.09)
DAPOS – depression	Lower	13.71 (4.04)	10.83 (4.32)
DAPOS – anxiety	Lower	8.24 (3.09)	6.75 (2.80)
DAPOS – positive outlook	Higher	8.71 (2.59)	9.67 (2.06)
WEMWBS (wellbeing)	Higher	38.59 (8.38)	42.92 (7.95)
MSK-HQ	Lower	31.71 (7.10)	27.58 (8.49)
4DSQ depression	Lower	4.24 (3.77)	3.50 (3.87)
4DSQ distress	Lower	20.05 (7.60)	16.5 (8.21)
4DSQ somatic	Lower	15.64 (6.72)	13.50 (6.80)
4DSQ anxiety	Lower	7.82 (6.51)	7.25 (6.80)

### Qualitative findings

**Figure 6.**
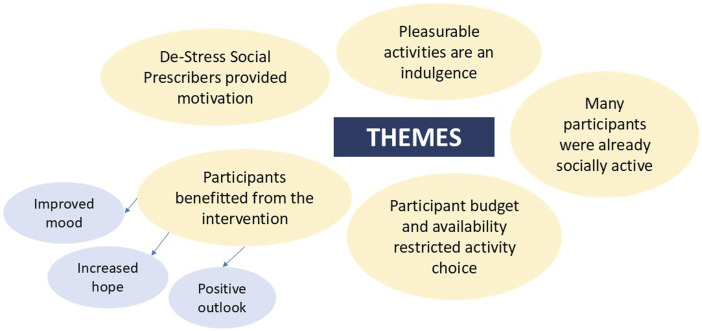
Key themes.

At baseline, participants displayed a strong belief in the relationship between pain and mood and supported the concept of pain-related distress as distinct from clinical depression. Five main themes were developed from analysis of follow-up data (Figure 6) and expanded in the text below.

## Participants reported benefit from the intervention

The majority of participants who engaged with the intervention reported positive changes due to taking part in the intervention, such as increased activity, improved mood, increased hope, and a new understanding of the benefits of doing things they enjoy. One participant described feeing stronger, healthier, and happier:

“I just feel stronger in myself… healthier in myself…I'm just happier as well, I'd like to say. I'm more open…” (P015, M)

Before the intervention, participants described feeling frustrated because they were unable to do the things they enjoy due to pain. The De-Stress SPs were able to discuss and address the barriers of these activities to find acceptable solutions. One participant described how a De-Stress SP session helped her see how alterations to the activities she enjoys could make them manageable, and in turn increased her feelings of hope:

“I do feel like I came out of that session with a lot more hope that actually there are so many things that I could alter so that I can do more. I hadn't thought of the alterations she'd suggested, so it gave me a bit of an, oh, there is something I can do about this.” (P004, F)

Although some participants described knowing that doing things they enjoy would be good for them, taking part in the intervention spurred a realisation that it is important to make time to do these things. Participation gave them permission to prioritise their wellbeing, and, when they started to re-engage, the importance of these activities was reinforced:

“I’m also realising that I do need to do those things [pleasurable activities], and if I don’t, it’s just long-term issues… I’m recognising that I've got to put that effort in because I know it makes a difference when I do.” (P009, F)

Once participants started participating in activities they enjoy, they reported that their attention was focussed more positively than before, and participants described a clear shift in mental wellbeing:

“Even though the pain still feels the same, and it still hurts the same, because my mind’s thinking about other things, it doesn’t feel like it’s hurting as much, so I haven’t had as many painkillers or anything, so that’s better. And my head feels clearer.” (P016, F)

## The De-Stress social prescribers supported motivation

Participants explained having someone to “report back to” helped them keep on track. The accountability this provided meant participants were more likely to push themselves into carrying through with their plans:

“Accountability to actually make yourself do it because we all know that these things are a really good idea, but actually having one-to-one support and saying, so what did you find? Have you done that? Come back to me and let me know how you found it. It’s like, okay, I’m going to have to do it then, aren’t I?” (P009, F)

Receiving the support from a real person (rather than online) was valued, and was reported to positively impact on engagement:

“I think it probably did help having an actual person. I think if I’d been given a website, I would have looked at it and gone, ‘that might have been a good idea, but I can’t really be bothered to do it.’ Then I knew I was going to speak to her and she was going to say, ‘oh, I’ve looked at all these things for you, what do you think?’ I wasn’t going to go, ‘well, I’ve not looked at anything you’ve sent me!’” (P004, F)

Participants described the De-Stress SPs with words such as “invested,” “understanding,” and “helpful,” and valued the rapport and relationships they formed, which in turn enhanced engagement.

## Participant budget and availability restricted activity choice

Many participants were restricted in the activities they chose due to cost, and some reported choosing activities that were free, rather than the activities they find pleasurable because of their financial constraints:

“it was the cost of things as well. That's the reason, really, that I went with the up-to-six-months gym membership thing that that was funded, whereas the pottery classes were going to be £30 a time.” (P005, F)

It was suggested that participant budget was considered before activities were suggested:

“the sessions need to focus on people with different income levels or looking into funding for things before they get offered to you.” (P005, F)

Another barrier to engagement was time; participants who were busy, for example with work or existing activities, found it difficult to engage in appointments with the De-Stress SP and adding activities to their schedule:

“The main thing is it's just been tough organising, trying to do an appointment…whether I can make it a certain time. I've been really busy with work.” (P007, F)

For those who worked shifts it was difficult to commit to a regular activity at a set time, and participants stressed the need for flexibility in the activities they chose.

## Some participants were already socially active

Participant interviews pre-intervention revealed that some (3 out of 16) agreed to take part because they wanted to help and facilitate understanding of pain-related distress, as opposed to feeling the intervention may benefit them personally. The De-Stress SPs questioned whether these participants were the population most in need of the intervention, as many were already socially engaged and active:

“I think it almost did feel a little bit like the patients that I was talking to were maybe a little bit too engaged in that they already have quite a lot of stuff going on…and slotting in other stuff did feel like it was a little bit difficult sometimes.” (SP002)

With these participants De-Stress SPs reflected that it was a struggle to know how to add anything:

“She talked about things she liked, and she liked a lot of things…she was the person that just tried to get on with life and try to put the pain to one side. Yes, she was involved in lots of local groups, committees. It was challenging in that I didn't really know what else I could offer.” (SP001)

Despite this, the De-Stress SPs reported finding ways to help such participants, for example, by supporting them to manage their time and prioritise the things that were most important and likely to benefit their wellbeing.

## Pleasurable activities are an indulgence

Some participants felt their responsibilities (e.g. work or caring for family) needed to take priority and viewed participating in pleasurable activities for themselves as an indulgence:

“I know how that sounds, but it's not a priority, my well-being. Even washing my hair is not a priority in comparison to making sure the kids have got their school uniform, have done their homework… It's really hard. I mean, if it was a doctor's appointment, that's quite a priority, you know, but because it's something just for me to try, it's an indulgence.” (P007, F)

This formed a barrier to engagement. Furthermore, understanding that doing pleasurable things is important and beneficial to wellbeing facilitated engagement. Participants who consciously made the decision to prioritise their wellbeing found the process beneficial:

“It’s been quite selfish in a way that I’ve had to look after myself now for a bit. My kids are 20 and 16, but it’s been quite nice for me to look after me for once… I’ve had to look after myself this time.” (P015, M)

## Discussion

### The acceptability of the intervention, and changes proposed

We have developed a novel intervention for those experiencing pain-related distress in primary care settings. This intervention (”De-Stress Pain”), delivered by social prescribers with an accompanying website, centres reactivation with experiences and activities patients enjoy and find pleasurable. It is based on a combination of ACT principles, behavioural activation and self-determination theory. The majority of participants in our proof-of-concept study found the De-Stress Pain acceptable and reported valuing this specific approach. Some reported positive changes due to participating in the intervention, such as improved mood, increased hope, a more positive outlook, and increased activity. Accountability appeared important for motivation, and participants appreciated live contact with a social prescriber. Some issues were described: Viewing an increase in pleasurable activities as an indulgence, or a luxury was identified as a barrier to engagement. Other barriers included participant availability due to other commitments such as work and family, and participant budget. Some participants recruited to this proof-of-concept study were already busy with social and pleasurable activities, suggesting further feasibility work is needed with more “deactivated” individuals.

Whilst participants were generally favourable toward the principle of the intervention, there are amendments required to increase inclusiveness and accessibility. Based on our findings, further work is needed to support patients in internalising the idea that focusing on and planning enjoyable experiences is a valid component of pain management. Individuals living with persistent pain are often supported through interventions that focus on pain management, medication optimisation, and increasing activity levels, with a strong emphasis on physical activity and psychologically informed approaches such as CBT and ACT.^34,35^ De-Stress Pain is intended to complement these evidence-based approaches by encouraging engagement in enjoyable and meaningful activities. The intervention is also strongly locally focused, which may enable it to support individuals in identifying appropriate and accessible opportunities within their communities to reduce pain-related distress, an area that can be challenging when services are delivered across broad regions. However, re-engaging in past hobbies may not align with some individuals’ expectations of what constitutes appropriate management of a clinical condition.

We also need to ensure that costs or physical access do not present barriers to engaging in enjoyable experiences. Being introduced to the theoretical underpinning of the intervention (that engagement in non-pain-related enjoyable experiences is beneficial), only to encounter financial or access barriers, may risk unintended negative effects. Given budget constraints, amendments are likely to focus more heavily on the role of social prescribers in supporting patients to identify free or low-cost, individualised, and accessible activities, alongside strategies to increase enjoyment and pleasurable experiences. Finally, we note that some participants were already active and routinely engaging in pleasurable activities. Further feasibility work is therefore required, particularly with patients for whom pain-related distress has led to reduced activity.

### Informing a feasibility trial

The proof-of-concept suggests that social prescribers are well-placed to deliver the intervention and that recruiting and training them would probably be feasible. Nonetheless, a feasibility study should explore other health care professionals who might deliver the intervention, to increase access, and optimise the fit with existing skills sets.

An additional strength of this work is that De-Stress Pain was specifically designed for delivery by social prescribers. While social prescribing is increasingly used to support people living with persistent pain, there is currently limited evidence describing interventions intentionally developed around the social prescriber role and skillset. By combining psychologically informed principles with a strong focus on local, meaningful, and enjoyable activity engagement, De-Stress Pain may represent a novel approach to supporting people experiencing pain-related distress within primary care and community settings.

While social prescribers have an advantage in their knowledge of local opportunities, they may need further training to deliver the intervention effectively, especially in reference to supporting self-reflection. De-Stress Pain provides social prescribers with training in the intervention principles and a support manual to guide delivery. However, more in-depth training on ACT principles and self-determination theory should be considered in future feasibility studies to optimise intervention delivery.

Another important lesson learnt from the POC is the need to develop a much broader and more representative recruitment. A feasibility study in future will inform on the viability of recruiting sufficient numbers into a trial. However, the current study has already indicated that recruitment should be from more diverse routes, for example, those who have completed pain management courses (representing more complex and enduring pain); those living with pain in populations under services and typically unrepresented in research, and unrepresented in our POC participants. Such groups include, for example, younger populations and people from minority ethnic backgrounds. Such omissions suggest that we should we cautious in concluding that the intervention is acceptable, as we can only conclude that is it acceptable to people recruited from primary care.

Optimising the length of the intervention should be a priority in a larger feasibility study. Future research should also consider how to integrate the intervention and its delivery with different existing pain services. Finally, future research might consider increasing access to the intervention by offering it on-line, but we note that our PPI were united in a strong insistence on in-person delivery.

### Strengths and limitations

Persistent MSK pain and the impact it has on a person varies widely. Using the PBA to guide development has resulted in a core product based on a rigorous transparent process, with clear guiding principles that can be adapted and updated according to the specific needs of the population. This flexibility means the intervention can be tailored according to factors such as participant mobility, pain location, ability to travel, and budget. De-Stress Pain also has flexibility in its implementation and could be adapted to a range of delivery contexts, including primary care, neighbourhood models of care within the NHS Long Term Plan, and partnerships with local charities, community organisations, and social care. Considering potential implementation pathways following a trial is an important component of the intervention’s acceptability and feasibility. PPIE involvement throughout the planning and development process ensured that decisions remained relevant to people living with MSK pain.

Due to the small sample size, and because over 25% of our sample did not provide data for the 12-week follow-up, the quantitative data has limitations. As a small proof-of-concept study we acknowledge the limited generalisability of our findings to the wider population, or to people with pain, that is, the sample was small, located in two geographical locations and the recruitment strategy did not attract an ethnically diverse sample. However, our sample was adequate to assess acceptability before rolling out to a larger sample. We also note that the intervention was developed with and for adults only: while conceptually De-Stress Pain is adaptable to support children living with pain who have withdrawn from activities, work would be needed with children, parents, siblings, and teachers.

A further limitation relates to outcome measurement. There is currently no validated standardised measure specifically for pain-related distress, which meant proxy measures and a single-item 0–10 pain-related distress rating were used in this proof-of-concept study. While these measures were considered adequate for exploratory purposes, future feasibility work should further examine the most appropriate way to capture changes in pain-related distress, including whether development or validation of a dedicated measure would be beneficial.

We have developed a novel approach to supporting people living with pain-related distress. We are not suggesting that De-Stress Pain should replace current evidence-based treatments; rather, further exploration is needed to understand how it may complement existing approaches. The results suggest promise, but also indicate where key amendments are needed. Larger feasibility studies of an amended intervention are needed with more diverse samples, before progressing to a full randomised controlled trial.

## Data Availability

The datasets generated during and analysed during the current study are available from the corresponding author on reasonable request.*

## Appendix 1

**Table A1.** Quotes to support findings in Table 1.

Findings	Supporting data	Intervention implications
1. General practitioners felt “stuck” when dealing with pain-related distress, and patients felt that there was little that could be offered to them by their GPs.	GP 6: “As GPs we want to help them but we’re struggling because there’s no resources. If you know the NICE guidelines which came last year about chronic pain management, clearly they said no to any analgesia for that matter. So again, we’ve limited resources to manage these kind of patients.” Person with pain (P5): “Well there was nothing I could do really, you know, nothing, I couldn’t do anything to improve it, you know, the only thing that flagged it was the painkillers.”	• Delivery outside of medical setting. • Not delivered by GPs.
2. GPs recognise the difference between depression and pain-related distress, and are therefore likely to be on board with the objectives of the intervention	GP 5: “Distress and depression, well I think… distress is when I think you know there’s something that’s happened specifically and the distress or the emotional upset is related directly to that thing.”	• Recruitment through primary care is viable.
3. GPs considered that social prescribers are ideally placed to encourage social reactivation, and can give more time than GPs.	GP 9: “The other thing we have is a social prescriber now within our practice and that’s really good. We haven’t got painkillers for these people. They don’t need surgery and they don’t need doctors. They need a life, basically…A lot of these people are very deconditioned and so I’m keen to get people exercising but I recognise that sending people to the gym is not really going to do it. So walking groups, gardening groups and that kind of thing or anything that gets patients socially engaged with their community.” GP 12: “Using social prescribers is good because they have more time per client than the GP, with the best will in the world we can’t spend an awful lot of time with any particular patient about emotional distress. Although obviously it’s our role to differentiate between that and true depression.”	• Delivery by social prescribers would fit within NHS systems and address intervention principles.
4. There was strong agreement amongst patients that a person delivering one-to-one contact was essential. The mode of contact mattered less.	Person with pain (P12): “If you just knew there was one person who was your case worker and they would deal with the GP, and the consultant, and they would be the person that says, ‘right, to get from here, what we need to do is just get it working.’ I think that would be beneficial…you just want one person, so you wouldn’t have to repeat the same thing 15 times”	• Participants need 1:1 contact with a person.
5. There was support for online information in addition to person-based delivery, but patients emphasised the information must be accessible to all.	Person with pain (P8): “Yeah that’s [both face-to-face and online] probably better. You know, you actually see somebody and speak to them, but do stuff in your own time as well. But you’ve got somebody explaining or just following up and just making sure that you’re doing what you’re supposed to.”	• Create a simple, user-friendly website, ensure use of lay language, images and ease of navigation.
6. Patients and PPIE told us that the most distressing aspects of living with pain were inability to do the things you used to enjoy, reduced social contact, and difficulty making plans.	Person with pain (P3): “it’s affected my well-being…not being able to do what I want to do. I used to be a runner and I can’t run anymore and that really has devastated me.” Person with pain (P8): “So normally trying to decide what time of day I’m going to do certain things knowing that I’m going to be in pain afterwards is difficult. You know and having to plan how you’re going to do everything, what you’re going to leave out, what doesn’t need doing, do I use my energy. It’s not even energy, but do I use my good part of the day to do something that I enjoy or something that I need to do? Yeah because doing the two isn’t easy.”	• Base the intervention on enjoyable activities. • Allow for opportunities to carry out some activities with other people. • Allow flexibility around approaching activities (no schedule, no use of the word ‘should’).
7. Addressing pain-related distress will take more than one consultation and continuity of care is important	GP 19: “I would say that’s the first and the most important thing and having continuity of care with the same doctor or the same clinician and not seeing a different person every time. It should be someone who really gets to know the patient and understands their point of view and the patient then, over time, gets to know and trust that doctor. I don’t think you can do it in one consultation.” (GP, male, aged 48 years)	• Offer more than a single session, and ensure sessions are with the same person.
8. Moving the focus away from pain is helpful	Person with pain (P13): “I suppose I’ve actually covered it myself really because it’s a sense of wellbeing. Things that I used to enjoy doing… I did used to be in a choir and that helped too… it’s something that takes you to a different place and stops you focusing on your pain. As I say, it will never take your pain away, obviously. Let’s say you go and sing in a choir for an hour, that’s an hour that you’re not thinking about your pain… That puts the pain a little bit out of my mind.”	• Don’t focus on pain.
9. Credibility is important	Person with pain (P04): “I think professionally doctors or people like you or research people, because you’ve got such a mind to do what you do, I think you can trust in what you’re saying…I would feel so much trust in anything that you would tell me because I know that you’re going to go to the right sources for things. So I think in that aspect professionals are invaluable because like arthritis research I’ll go to them because I know they’re up to the minute with all the research that’s coming out and I can feel confident in what I’m reading. So I think yeah, a person who is specialising in something and you know that that’s their passion and that’s what they do, you can trust in it. You can feel completely relaxed. I know I can go to this and it will tell me what I need to do or hear.”	• Intervention needs to be delivered by experts in social reactivation.
